# Motivation of Owners to Purchase Pedigree Cats, with Specific Focus on the Acquisition of Brachycephalic Cats

**DOI:** 10.3390/ani9070394

**Published:** 2019-06-27

**Authors:** Liran Plitman, Petra Černá, Mark J. Farnworth, Rowena M.A. Packer, Danièlle A. Gunn-Moore

**Affiliations:** 1 Royal (Dick) School of Veterinary Studies and The Roslin Institute, The University of Edinburgh, Easter Bush Campus, Roslin EH25 9RG, Scotland; 2 Department of Veterinary Services, Ministry of Agriculture and Rural Affairs, Animal Welfare Unit, Beit Dagan 5025001, Israel; 3 University of Veterinary and Pharmaceutical Sciences, Brno 612 42, Czech Republic; 4 School of Animal, Rural and Environmental Sciences, Nottingham Trent University, Southwell, Nottinghamshire NG25 0QF, UK; 5 Royal Veterinary College, Department of Clinical Science and Services (CSS), Hatfield AL9 7TA, UK

**Keywords:** cat acquisition, brachycephaly, selective breeding, conformational disorders

## Abstract

**Simple Summary:**

Selective breeding of domestic cats (*Felis catus*) has resulted in a variety of temperaments, body shapes, facial features and, in particular, coat types and colours. There are now over 70 recognised cat breeds, created over 150 years. Many cat owners select their cat for features that appeal to them, so cat breeders primarily focus on these aesthetic traits, changing cat features to their concept of beauty or appeal. Unfortunately, these changes can impact heavily upon cat health and welfare, as inbreeding and breeding for extreme physical conformations has become prevalent. Particular concern focuses on brachycephalic (BC) cats with wide flat faces (e.g., Persians and Exotic Shorthairs). Using a questionnaire for cat owners, we identified marked differences in how owners went about acquiring their cats when we compared owners of BC cats, pedigree (P), (but not BC) cats and non-pedigree (NP) cats; 1367 responses (BC *n* = 85, P *n* = 400, NP *n* = 882). Owners of BC cats were less likely to undertake significant research before buying their cat (e.g., not looking into management requirements or potential health problems associated with the BC face-shape and/or Persian coat) compared to P owners. Once owned, they did not consider their cat as healthy (especially related to eye and skin conditions) compared to P owners. BC owners were also less likely to recommend their breed to others, possibly reflecting poor health experiences and/or management issues.

**Abstract:**

Background: Cats are globally popular pets and pedigree cats are increasingly prevalent, with brachycephalic breeds being the most registered breeds. How owners decide upon and acquire their cats is poorly understood. Moreover, there are growing concerns about the health and welfare of brachycephalic (BC) dogs and recent studies are raising the awareness of health and welfare problems in BC cats. Methods: An online survey investigated owners’ motivations, perceptions and behaviours prior to, during and following acquisition of non-pedigree (NP), extreme brachycephalic pedigree (BC; i.e., Persian and Exotic Shorthair) and mild to non-BC pedigree (P) cats. *Results*: The survey received 1367 valid responses (NP *n* = 882, P *n* = 400, BC *n* = 85 (6.2%)). There were marked differences between NP, P and BC owners’ perception of their cats’ health and welfare, reason(s) for acquisition and its process. Owners of NP were less influenced by appearance, behaviour and other features than P or BC owners. In contrast, P and BC owners were highly influenced by appearance, with P owners also placing greater importance on good breed health than BC owners. BC owners were less likely to recommend their breeds to prospective cat owners, apparently concerned by high maintenance requirements. *Conclusion*: Further research is needed to determine how decision-making is constructed and how it may be improved, especially in respect of welfare outcomes for extreme BC cats given the increased weighting given to appearance over health.

## 1. Introduction

Domestication of cats began ~10,000 years ago, with selection for cats having a more docile nature [1]; these notoriously shy solitary hunters had to adapt to living in close proximity with people, and with other cats [2,3,4]. People may also have selected for rodent-catching abilities and favourable coat colours [5] (e.g., the earliest documented ‘cat show’ was held in England in 1598 and awarded titles such as ‘best mouser’ [6]). Unlike dogs, that have been artificially selected for a range of complex behaviours (such as herding sheep, guarding livestock and a variety of hunting behaviours), cat breeding has focused primarily on aesthetic traits [2,7]. This has resulted in different coat colours, patterns, textures and lengths, changing facial features and body shapes [1,6].

Domestic cats (*Felis catus*) are now more popular than dogs; there are ~80 million pet cats and ~70 million pet dogs in the USA, and 11.1 million pet cats and 8.9 million pet dogs in the UK [8,9,10]. Pedigree cats now account for 16%–18% of pet cats in the USA and 8%–11% in the UK [8,11,12]. The breed standards, or ‘the standard of points of excellence and beauty’ that define each breed were introduced at the first modern cat show in London in 1871 [6]. They are set by ‘cat fancy’ organisations, which are breed registration bodies involved in showing, promoting and breeding cats (e.g., Cat Fanciers’ Association (CFA), The Governing Council of the Cat Fancy (GCCF), Fédération Internationale Féline (FIFe) and The International Cat Association (TICA)). These associations now recognise over 70 cat breeds worldwide [13], each being defined by exacting breed standards that dictate their ideal body shape, size, colour and proportions. Extreme phenotypes defining some pedigree breeds can impact heavily upon feline health and welfare [14].

‘Favourable’ features, when integrated into breed standards, can become more pronounced with time as breeders exaggerate them, with the ethos of ‘if something is desirable, then more must be better’ [6]. Breeders often use selective breeding from a restricted pool of cats to ensure conformity with breed standards. This can result in inherited disorders due to recessive mutations in a relatively small gene pool [14].

**Figure 1:**
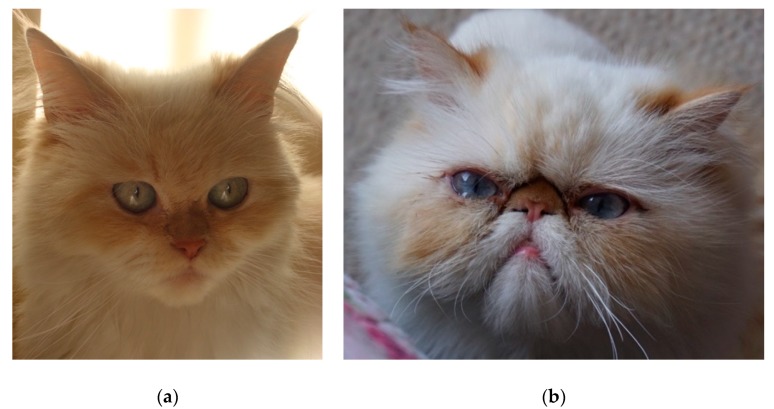
Traditional (´doll-face´) (**a**) vs. modern type (´peke-face´) (**b**) Persian cat.

An example of exaggerated physical features is brachycephaly (i.e., a short muzzle and flat face), seen in dog breeds such as Pugs, Pekingese, French and English Bulldogs, and cat breeds such as Persian (PER) and Exotic Shorthair (EXO) cats [15,16]). While brachycephalic (BC) breeds are not a recent creation, (pugs were recognised the 16^th^ century [17], and Persians are one of the oldest known cat breeds [2]), modern versions are considered much more extreme. Persians initially had only mild BC features (now known as ‘doll-face’ Persians), but selective breeding has resulted in ‘peke-face’ Persians, with extreme BC features (Figure 1). The CFA now consider the ‘peke-face’ to be the modern Persian standard [18], hence it has gained favour among pet owners and breeders [11,19]. Persian cats were at the peak of their popularity in the USA between 1985–1995 with over 1000 kittens registered by TICA every year; however, their popularity has decreased in the past decade, with 400–600 kittens registered in 2018 [20]. Exotic Shorthairs are now the most frequently registered cat breed in the USA, while in the UK it is the British Shorthair (also brachycephalic) followed by the Persian [11,19]. The BC phenotype carries with it the risk of ophthalmic, facial, dental, respiratory, neurological and reproductive problems, which are more severe with the more extreme phenotype [21,22,23]. 

The reasons for acquiring a particular type of cat (i.e., non-pedigree, pedigree or a particular breed) may affect the cat’s health and welfare, and the owners’ overall satisfaction with their pet. Certainly, pedigree dog ownership, including that of non-BC (NBC) and BC breeds, has often been shown to be driven by different factors, including differential emphases on appearance and health [24]. However, the reasons and methods for cat acquisition and ownership, especially the motivations for acquiring a pedigree cat, have been studied only rarely. To date, most studies have focused on identifying motivating factors with the aim of improving rehoming from rescues centres [9,25,26], for example, the annual PDSA (People´s Dispensary for Sick Animals) PAW (PDSA Animal Wellbeing) Report in the UK asks respondents about research undertaken prior to acquisition, and where their cat came from [9].

In dogs, studies have found that complex behaviours (e.g., working abilities such as the ability to herd sheep), longevity and health are not priorities for most potential owners; they are more often attracted to pedigree dogs because of appearance and current fashion trends [9,27,28]. This is particularly apparent with BC breeds [24]. BC owners have been found to be more influenced by their chosen breed’s appearance over its health or longevity compared to non-BC owners. In addition, BC owners were less inclined to follow recommended responsible purchasing behaviours, including not asking their breeder for the dam or sires health records, sourcing dogs from online sources, and buying their puppy the first time they met it [24]. These behaviours may impact the individual dog’s welfare and owners’ satisfaction with their new dog. In addition, such behaviours may support harmful breeding practices, where genetic testing for inherited diseases is not performed, animals may be kept in poor conditions (e.g., puppy farms), and breeding is undertaken solely for financial gain [8,29].

Similar data for cats is not yet available. Thus, the aim of this study was to examine the motivations and behaviour of people when acquiring a cat. We hypothesised that owners’ behaviours and motivations would differ depending upon breed choice and whether or not they were acquiring a pedigree cat. A particular focus considered whether they were acquiring a BC or non-BC pedigree.

## 2. Materials and Methods

### 2.1. Survey Design and Distribution

The survey was adapted from a similar questionnaire that investigated acquisition motivations and behaviour in owners of popular pedigree dog breeds [24]. It was adapted to meet the needs of cat owners and designed to gather information on owners’ motivations, perceptions, behaviour and knowledge prior to, during and after acquiring their cat. These data were then examined in relation to three groups of owners:

(1) Owners of non-pedigree (NP) cats
(2) Owners of pedigree (P) cats (including all pedigree cats and wildcat hybrids, other than extreme BC breeds; e.g., Persian and Exotic Shorthair cats)
(3) Extreme BC breeds (e.g., Persian and Exotic Shorthair cats)

The BC breeds (e.g., Persian and Exotic Shorthair cats) were separated from the other P cats in our study as brachycephalic breed-types such as Persians or Exotics showed the greatest divergence for NP cat skull morphology [21].

The survey (see Appendix A) was composed of seven sections:

(1) Participants (owners) demographics: e.g., country of residence; age; gender; level of education; living area; marital status; whether they were keeping other pets; whether they were first time cat owners.
(2) Cat demographics: e.g., type/breed; age; gender; neutering status; outdoor access.
(3) Reasons and motivations for acquiring the type of cat: e.g., factors in acquiring a specific type/breed of cat (appearance, popularity, behaviour, health, etc.); general motivations for acquiring a cat (companionship, for breeding, etc.).
(4) Pre-acquisition research and knowledge: e.g., quantity and quality of research pertaining to type/breed of cat; knowledge of the cat’s type/breed prior to and following research (health, behaviour, lifespan, etc.).
(5) The acquisition itself and the new owners’ requests: e.g., how the cat was acquired (via charity organisation, breeder, etc.), and owner behaviour at acquisition (e.g., asking to see health records, seeing the cat’s parents/siblings, etc.).
(6) Post-acquisition perspectives: e.g., owners’ satisfaction with their cat; whether they would recommend the type/breed of their cat to others.
(7) Cat’s health and owners’ perception of its welfare: e.g., reasons for taking the cat to the vet; diagnosed health problems; owners’ perception of cat’s quality of life.

The survey was hosted on the Bristol Online Survey (BOS) platform, and participants were approached via social media. The survey was approved by the Human Ethical Review Committee (HERC) at the Royal (Dick) School of Veterinary Studies.

### 2.2. Statistical Analysis

Data analyses were performed in IBM SPSS Statistics package v23 (SPSS Inc, Chicago, IL, USA). Descriptive statistics were used to describe general respondent and feline demographics, with medians and 25–75th percentiles presented for non-normally distributed data. Contingency tables were created to examine the relationship between categorical variables. Chi-squared test of independence was used to analyse categorical data (e.g., associations between owner/cat demographics and type/breed of cat) and ordinal data (e.g., how did owners perceive different features of their cat on a scale of 1–3). The Fisher’s exact test was conducted when chi-squared contingency tables had cells with expected counts less than five.

Post hoc analysis was performed on chi-squared outputs to determine where significant differences actually existed and to control for type-I errors. This was done by comparing adjusted residuals and using the Bonferroni correction to the alpha level [30]. The adjusted *p*-value was then reported instead of the raw *p*-value, which was set at 0.05. When Fisher’s exact test could not be performed on significant chi-squared outputs due to limitations of the software, categorical/ordinal data were revised (e.g., Likert scales were reduced from 1–5 by condensing categories 1–2 and 4–5). When data could not be revised, results of the chi-squared test were not reported, and descriptive statistics were given instead. The Kruskal–Wallis test for one-way analysis of variance by ranks was performed to determine the effect of cat type on the health score given by owners.

## 3. Results

### 3.1. Survey Overview and Demographics

Survey data were collected between June 18 and July 30, 2017. A total of 1434 responses were received, of which 67 incomplete responses were discarded, resulting in 1367 responses being included in the data analysis. The majority of respondents were female (87.4%) (Table 1), with 43% residing in the UK and 45% being young adults (18–34 years of age). Most respondents did not live with children (77.4%) and had higher levels of education (60.7%), with the most common household income being £25,000–£49,999 (27%), followed by £10,000–£24,999 (18.7%). Over a third (37.8%) of participants were married, followed by in a relationship (29.2%) and single (24.9%). Most owners (71.8%) lived in a house or a semi-detached house, which was typically suburban (41.1%) or urban (35.3%). Most owners (82.8%) had kept cats before the one they were completing the survey for, and (60%) had no other pets. 

Of the 1367 responses, 882 were for non-pedigree (NP) cats (64.5%), 400 were for pedigree (P) (excluding extreme BC cat breeds) (29.3%) (Table 2) and 85 were extreme BC breeds (i.e., Persian and Exotic Shorthair; 6.2%). When looking at all BC breeds, this included British Shorthair, Burmese, Exotic Shorthair, Persian and Scottish Fold (*n* = 169, 12.4%), but given the variation in facial morphology from the NP in some breeds (e.g., Persian and Exotic Shorthair cats), only these two extreme BC cat breeds were analysed separately; the mildly BC breeds, (e.g., British Shorthair, Burmese, etc.) where analysed with the other pedigree cats.

The most common age group of cats was 0–3 years old (39.1%), followed by 3–6 years (25.8%). The cats were equally divided between males and females, and most (82.8%) were neutered. While most cats had some access outdoors (either always (15.8%), at certain times (19.6%) or to a restricted area (19.3%)), over a third (35.4%) had no access outside.

### 3.2. Characterisation of Owners by Cat Type

Both NP and P owners were most commonly from the UK (42.9% and 47.8%, respectively) (Table 3), while most BC owners were from the USA (44.7%), (*χ ^2^*= 27.35, DF = 4, *p*-adj. = 0.005). The major age groups were 25–34 years and 45–54 years, with NP owners most commonly being 25–34 years (31.3%), and P and BC owners most commonly being 45–54 years (26.3% and 31.8%, respectively). NP owners were significantly younger than P and BC owners, with many being 18–24 years (21.5%) (*χ^2^* = 107.89, DF = 8, *p*-adj. = 0.003), while BC and P owners were more likely to be 45–54 years, with many P owners being over 55 years (15.3%) (*χ^2^* = 107.89, DF = 8, *p*-adj. = 0.003). The majority of owners were female, with no difference between the NP, P and BC groups (*p* = 0.06).

The majority of owners had attended higher education, with no difference between groups (*p* = 0.54). Most owners were ‘Married’, followed by being ‘In a relationship’ for NP and P owners (32.1% and 25.3%, respectively), and ‘Single’ for BC owners (28.2%). Pedigree owners, although the majority of them are in a relationship, are more likely to be single, compared to NP and BC (*χ^2^* = 10.01, DF = 2, *p*-adj = 0.005)

The majority of owners lived in a house/semi-detached house, with more than 40% of NP and P owners living in a suburban area and 42.4% of BC owners living in an urban area; however, there was no difference in living area or type of residence between groups (*p* = 0.07, *p* = 0.18, respectively).

Most owners had owned a cat before, but a significant difference was found between NP and P owners, with more NP owners being first-time owners (*χ^2^* = 34.01, DF = 2, *p*-adj. = 0.008). The majority of owners acquired their cat when it was aged 0–1 year, their cat’s current age was mostly 0–6 years, and there was no difference in gender distribution between groups (*p* = 0.70). Although the majority of all cat groups were neutered, NP cats were more likely to be neutered and P cats were less likely to be neutered (*χ^2^* = 140.60, DF = 2, *p*-adj. = 0.008); however, there was no significant difference in the BC group (*p* = 0.63).

The majority of NP (66.3%) and P (65.7%) cats had some outdoor access; however, most BC cats were not given outdoor access (57.6%) which was significantly higher than in the NP and P groups (Fisher’s exact test = 19.87, *p*-adj. = 0.005).

### 3.3. Owners’ Motivations and Perceptions

There was a statistically significant association between cat type and their owner’s scoring (on a scale of 1–3, 1 is ‘poor’ to 3 is ‘good’) on different aspects of their cat’s appearance, behaviour, companionship, ease of maintenance, health, energy level and cost (Table 4a). The majority of owners gave the highest scores to appearance, behaviour, companionship, ease of maintenance, health and energy level (median = 3 (25–75th percentile: 3–3)), followed by a moderate score for cost (median = 2 (25–75th percentile: 2–3)). Owners of NP and P cats gave the highest scores for appearance, behaviour, companionship, ease of maintenance, health (median = 3 (25th–75th percentile: 3–3)) and energy level (NP median = 3 (25th–75th percentile: 2–3); P median = 3 (25th–75th percentile: 3–3)), followed by a moderate score for cost (NP median = 2 (25–75th percentile: 1–3); P median = 2 (25–75th percentile: 2–3)). Owners of BC cats gave the highest score for appearance, behaviour, companionship, health (median = 3 (25–75th percentile: 3–3)) and energy level (median = 3 (25–75th percentile: 2–3))*,* but scored lower on ease of maintenance (median = 2 (25–75th percentile: 1–3)), and higher on cost (median = 3 (25–75th percentile: 2–3), Table 4b.

A statistically significant relationship was also found between cat type and the scoring given to different factors that influenced the owners in their choice of cat (on a scale of 1–3, 1 is ‘little or no influence’ to 3 is ‘strong influence’) (Table 5a). The highest scores across all types of owners were given to appearance and companionship (median = 2 (25–75th percentile: 1–3)). NP owners scored lower (median = 1) on appearance (25–75th percentile: 1–3), health (25th–75th percentile: 1–2)*,* companionship (25–75th percentile: 1–3)*,* popularity (25–75th percentile: 1–1), celebrity endorsement (25–75th percentile: 1–1)*,* cost (25–75th percentile: 1–2) and ease of maintenance (25–75th percentile: 1–3) (Table 5b). P owners scored highly (median = 3) on appearance (25–75th percentile: 3–3), health (25th–75th percentile: 1–3) and companionship (25–75th percentile: 3–3) (Table 5b). BC owners scored highly (median = 3 (25–75th percentile: 2–3) on appearance and companionship only (Table 5b). There was no significant relationship between the scoring given to costs and cat type/breed (Table 5a).

The majority of owners gave the highest score in satisfaction with their cat and its breed/type (on a scale of 1–3, 1 is ‘unhappy’ to 3 is ‘happy’; median = 3 (25–75th percentile: 3-3)) (Table 6). There was no significant relationship between owner’s satisfaction with their cat and its breed/type (*p* = 0.57). However, a significant difference was found between owners of different groups regarding whether they would recommend their breed/type of cat (Fisher’s exact test (FET) = 19.8, *p*-adj. = 0.008). Owners of BC breeds were less likely to recommend their breed of cat, compared to the owners of P and NP cats. Owners were asked to comment in free text as to why they would or would not recommend their particular type of cat to others; a substantial number of BC owners noted significant maintenance issues, such as needing to groom long hair (in Persians) and to clean eyes frequently (in Persians and Exotic Shorthairs).

### 3.4. Owners’ Behaviour Prior to and During Acquisition

Owners of NP cats were less likely to conduct research prior to acquisition of their cat compared to P and BC owners (*χ^2^* = 698.2, DF = 6, *p*-adj. = 0.004) (Table 7). Differences were also identified in information sourcing; the research source most commonly used by all groups was online research/websites, with no significant difference found between groups (*p* = 0.34). However, P owners were more likely to talk to breeders as a source of research (FET = 29.9, *p*-adj. = 0.003), while NP owners were more likely to talk to a veterinary professional (FET = 16.4, *p*-adj. = 0.027).

The most common method of acquiring a NP cat was from a charity rescue shelter (37.5%), followed by friend/neighbour (27.7%) and by finding/rescuing it (19.4%) (Table 8). Among the other owners, 71% of P owners and 54.1% of BC owners purchased their cat from a breeder. There was a significant difference between the groups in the acquisition of their cats (*χ^2^* = 788.7, DF = 12, *p*-adj. = 0.001). P and BC owners were more likely to acquire their cat from a breeder than NP owners; however, BC owners were less likely to acquire their cat from a breeder than P owners (*χ^2^* = 20.7, DF = 5, *p*-adj = 0.004), which could indicate a problem of BC cats being purchases from non-registered breeders rather than breed clubs, potentially moving towards even more extreme phenotypes. NP owners were more likely to acquire their cat from a charity/rescue shelter, friend/neighbour or to find/rescue their cat compared to P owners. Moreover, NP owners were less likely to have acquired their cat by breeding the cat themselves compared to P owners. When asked how they found the breeder, the most common answer among P and BC owners was through the breeder’s website (21.7% and 28.3%, respectively), followed by a recommendation from another breeder or at a cat show (18.5% and 15.2%, respectively) and via breed club registry (14%, P owners) and pet selling websites (e.g., Gumtree) and social media (13%, BC owners) (Table 9).

Significant differences were found between P and BC owners regarding the questions they asked prior to acquisition, and the information they gained about their cats. BC cats were less likely to be born naturally (e.g., they were more likely to be born by caesarean) and BC owners were less likely to know how their cat was born compared to P owners (FET = 10.4, *p*-adj. = 0.008). Owners of BC cats were less likely to have had both parents of their cat undergo tests prior to breeding (e.g., genetic testing, hip radiographs, heart scans), and be less likely to know if either or both parents had undergone testing compared to P owners (FET = 10.6, *p*-adj. = 0.006) (Table 10).

### 3.5. Cats’ Health and Welfare

Owners were asked to give a health score for their cat, depending on how healthy they considered their cat to be over the course of his/her life (with 1 being very poor health and 7 being very healthy). The 1–7 Likert scale was adapted from the Murphy et al. dog questionnaire [31]. The Kruskal–Wallis test was used to determine the effect of cat type on the health score given by owners. A significant difference was found between the cat groups and health score given by owners (H = 22.7, DF = 2, *p* < 0.001); BC cats received the lowest score, while P cats received the highest score. The median health score given to NP and P cats was 7 (25–75th percentile: 6–7), compared to a median of 6 (25–75th percentile: 5–7) for BC cats. The BC cats had more owner-reported problems affecting their eyes (*χ^2^* = 19.4, DF = 2, *p*-adj. = 0.008) as well as more owner-reported heart and blood vessel problems (Fisher’s exact test = 5.855, DF = 2, *p*-adj = 0.008) compared with NP and P cats, and appeared to be less healthy than NP and P in most health categories (glands, skin and/or hair, muscles and/or skeleton, reproductive organs, the urinary system and breathing), with eye, heart and blood vessel problems being significant. BC cats also appeared to have more urinary tract problems, which could be related to predominantly indoor housing; however, more research is needed in this area [32] (Table 11).

In general, the majority of owners scored low on the effect they thought any health problems their cat had was having on its quality of life (QoL) (median = 1, i.e., cat’s health problems ‘have little or no effect on their quality of life’ to 5 ‘have a significant negative effect on their quality of life’). However, BC owners were more likely to score highest (i.e., ‘have a significant negative effect on the cat’s quality of life’) for problems related to skin and/or hair (FET = 9.9, *p*-adj. = 0.005). There were no differences regarding all other health problems.

## 4. Discussion

This paper expands our knowledge about owner motivation for obtaining a cat, their opinions and views following acquisition, and their perceptions of their cat’s health and welfare. This is important; while cats are favourite pets worldwide, few studies have previously investigated how and why they are acquired [9,25,26,33]. This is concerning, especially regarding the purchase of pedigree cats, which make up a significant percentage of the pet cat population, and of BC cats in particular, since they are now the most popular cat breeds in the USA and the UK [8,11,12,19].

Our findings suggest that there are marked differences between NP and pedigree (including BC) cat owners, as well as between BC and P cat owners in their motivations for acquiring a cat, their pre-acquisition knowledge and research, behaviour during purchase, owners’ satisfaction and perceptions of their cat’s health. Importantly, BC owners were less likely to undertake significant research before acquiring their cats, less likely to consider their cat as healthy (especially related to ocular and skin conditions, although still giving a generally high score) and less likely to recommend a BC cat to others. That BC owners were less likely to undertake significant research might mean that they were unaware of the health problems connected with the BC breeds (such as breathing difficulty, skin and ocular diseases) before they acquired their cat, and therefore why they were, ultimately, less likely to recommend a BC cat to others. This differs from BC dog owners, where in one study, the majority of respondents would recommend their breed to a friend or family member and there were no differences in attitude between BC and non-BC owners [24]. However, that BC owners are less likely to consider their cat as healthy suggests that they do have some awareness of their cat’s health problems. On the other hand, BC owners were older than NP owners and less often first-time cat owners, which differs to that reported for owners of BC dogs, who were younger, more likely to live with children, and to be buying their chosen breed for the first time than owners of P or NP dogs [24].

The general profile of the owners in this study was predominantly female, well educated, unmarried (i.e., ‘single’ or ‘in a relationship’), with no children or other pets, and they had owned cats before. These findings are largely compatible with past surveys [34,35,36,37,38]. However, previous choices for ‘marital status’ were binary (either ‘single’ or ‘married’) [34,38], while the current respondents could also choose ‘in a relationship’, which many did, helping to debunk the cultural archetype of cat owners as lonely single women [39]. However, it also seems that women are often more likely to respond to surveys [40]. The predominance of experienced owners (i.e., not first-time owners) may indicate their representation in social media cat-related groups, and/or their greater willingness to reply to surveys regarding cats [41].

The main differences in owner characteristics between owners of NP and pedigree (including BC) cats were age and, to a less extent, first-time cat ownership. The results suggest two main age groups among cat owners (i.e., 25–34 and 45–54 years), with NP owners more likely to be younger than P and BC owners. In contrast, a similar study exploring dog ownership found that BC dog owners were younger than non-BC pedigree owners [24], where it was suggested that young owners were more likely to be influenced by social media into buying a BC breed of dog. Although BC cats (and other pedigrees) are well represented in social media and are celebrity favourites [42], media exposure does not appear to play as significant a role when influencing the purchase of BC cats as it does with BC dogs. It may be partly influenced by NP cats being more popular and ‘mainstream’ than NP dogs; only ~10%–20% of pet cats are pedigree in the UK and USA, compared to ~60%–75% purebred dogs in the UK and USA [8,9,11]. However, Facebook (where the survey was distributed) tends to attract young adults (18–34 years) [43], and while our largest age range was 25–34 years, many of our P and BC owners were in the 45–54 years category, and some were over 55 years, so this age difference appears to be real.

While the majority of owners in the current study were experienced cat owners, NP cat owners were more likely to be first-time owners compared to the other groups. This may be attributed to the more spontaneous nature of acquiring a NP cat, typically by rescuing it as a stray or by the cat ‘adopting’ the owner, without the owner necessarily intending to own a cat [34,44,45]. In comparison, to acquire a pedigree cat, owners are required to be more proactive, deciding on a specific breed, and the acquisition itself may require a more comprehensive process (i.e., being more likely to need pre-purchase visits to breeders, breed-specific rescues, or the need to save up to afford a particular breed). That said, some adopters may make multiple visits to shelters prior to adopting a NP cat.

Owners of BC cats differed from P and NP owners by country of residence. That many BC owners lived in the USA may be attributed to the survey’s distribution bias towards American cat fanciers’ Facebook groups (e.g., the CFA) in that they are the largest registries of pedigreed cats (which will have selected for all pedigree cats) and, in particular, to the ever-growing popularity of BC breeds in the USA, where Exotic Shorthair cats have been first place for three consecutive years and Persian cats are continually in the top five [46]. The same trend can be seen in registration of BC dogs as the number of registered French Bulldogs by The Kennel Club increased from 1521 in 2009 to 36,785 in 2018 [47].

The majority of the cats in all three groups were neutered. However, P cats were least likely to be neutered which might suggest some P cats in the study were purchased for breeding. Owners further differed in the outdoor access they gave their cat. The majority of BC owners gave their cat no outdoor access (compared to P and NP owners); the CFA recommends keeping Persian cats indoors [48]. One study found that Persian cats had the lowest activity level of 17 cat breeds studied, which may have been influenced by their limited access to the outdoors [49]. Similarly, the current study found that BC owners scored their cats’ energy and exercise levels lower than the owners of other cats. These findings could also explain why BC cats were not let outdoors, as BC owners may perceive that their cats have a reduced need for outdoor exercise. However, what appears to be BC cats’ predisposition to low activity levels, could actually reflect their inability to lead normally active lives because of conformation-related health problems (i.e., difficulty breathing during exercise due to conformational obstruction of the upper airways and thermoregulatory problems) [21,50,51]. Similar findings for BC dogs have also been described [52,53,54].

The most important reasons for acquiring a cat were given as its appearance and its likelihood to be a good companion. This agrees with previous research on cat ownership [33,44]. Owners of NP cats were the least likely to acquire their cat for any specific reason regarding its physical type. A study on BC versus other pedigree dog owners found significant differences in owner motivation between these groups, with BC dog owners being most motivated by appearance, followed by breed size suited to lifestyle, good dog breed for children and good companion breed [24]. In the current study, the primary motivations were generally similar among BC and P cat owners as they were highly influenced by their breed’s appearance, and its perception as a good companion breed. Appearance-driven breeding is acknowledged to be problematic for health in dogs and cats [52,55], and thus it is of concern that appearance is a primary motivation in owners acquiring cats.

A majority of pedigree owners also chose ‘good health’ as a major influence in acquiring their breed of cat. This may reflect the desire to find the most ‘healthy’ pedigree cat that they can, or that they are uninformed about their breeds’ disposition to inherited and/or conformational disorders. However, most BC owners did not choose perceived good health as an influence in acquiring their cat. This could indicate that these owners are uninterested in the known health issues of these breeds, are aware of the breed’s health problems but do not let them influence their choice or believe that significant health problems are ‘normal for breed’ [52]. That BC owners scored lower on the perception of the health of their cat than NP and P owners (although still giving a generally high score) suggests that BC owners have some awareness of their cat’s health problems, but perhaps underestimate their severity or impact on QoL and thus still opt to acquire these breeds.

Owners of NP and P cats were more likely to recommend their type of cat than BC owners were. This may be explained by a number of results (i.e., NP owners scored highest on ease of maintenance and lowest on costs, suggesting their satisfaction may be due to the easy management of their cats). However, BC cat owners, while scoring highly on satisfaction level, were least likely to recommend their breed. When asked why they would not do so, many respondents mentioned significant maintenance issues, such as needing to groom long hair (in Persians) and to clean eyes frequently (in Persians and Exotic Shorthairs) as problems. Of note, owners who mentioned having to clean their cat’s eyes, referred to this as a maintenance problem rather than a health problem. However, chronic epiphora is a significant health problem in BC breeds, where the brachycephalic conformation results in large flat eye sockets, kinking of the nasolacrimal ducts and poor tear drainage, incurring the risk of significant facial and ocular problems, including facial irritation and infection, non-healing corneal ulcers and corneal sequestra, conjunctivitis and entropion, in severe cases necessitating enucleation [23,56,57,58,59,60]. Unfortunately, while many BC breed clubs recommend regular face washes as part of routine care [15,16,47], they do not regard it as a conformational problem that needs to be dealt with at the breed level.

While breed clubs may deny these health issues, previous studies have reported significant health risks in Persian cats related to their breed-specific physiognomy (i.e., dermatological, ophthalmological and dental disorders [22,23]). The current study found BC cats to have more owner-reported health problems in almost all body systems than the other cats, with those affecting the ‘eyes’, and ‘skin and/or hair problems’ to affect the cats’ QoL most. Whether the latter referred to infected facial skin folds or to problems regarding the coat (e.g., excessive matting) is unclear and requires further investigation. The findings of the current study regarding the health of BC cats proves worrying as BC owners scored lower on their perceived healthiness of their breed compared to other owners, but still chose an overall high score for satisfaction with their breed. This raises a question as to whether BC owners have some awareness of their cat’s health problems and perhaps underestimate their severity or impact on QoL and thus still opt to acquire these breeds despite reporting significantly more health issues than other owners. It appears that BC owners are aware of their breed’s health problems (at least to some extent) but underestimate their severity or fail to make the connection of their impact on the cat’s welfare (as has been reported in owners of BC dogs [52]).

Not surprisingly, the different cats were acquired from differing sources, with most NP cats acquired from charity rescue shelters, friends/neighbours or by finding/rescuing it, all with similar frequencies as seen in the recent PAW Report [9]. While most P and BC cats were purchased from a breeder, with the breeder having been identified through their own website, following a recommendation from another breeder, or at a cat show, more BC cats than P cats were acquired through websites selling pets (e.g., Gumtree) or via social media. This is a concern as these sources are poorly regulated, and this may reflect BC cats being sold by unscrupulous breeders, and/or being ‘dumped’ when found to be unhealthy and/or requiring more effort than a new owner is willing to undertake [61]. This may reflect a similar situation seen with French Bulldogs (a BC dog breed) in the UK, where the RSPCA (Royal Society for the Prevention of Cruelty to Animals) is seeing more of this breed being breed on puppy farms, illegally imported, or dumped (e.g., at Battersea Dogs Home) [62,63].

The amount of research undertaken before acquiring their cat varied between the groups, with NP owners undertaking little compared to P and BC owners. This supports the idea that NP cat acquisition is much less premeditated than that for pedigree cats, and perhaps more likely to result from a situation of immediacy [9,33]. Of interest, NP owners were more likely to seek out a veterinary professional to learn about cat ownership, while pedigree owners preferred turning to a breeder to research breeds and ownership. This may help explain the finding that pedigree owners, perhaps unexpectedly, often chose ‘good health’ as a significant influence in acquiring their breed. It can be presumed that owners who research the health of a certain breed by talking only with a breeder, and not with a veterinary professional, may not receive the full scope of the potential breed-related health problems. Within the pedigree owners surveyed, significant differences were found between P and BC owners regarding the questions they asked before acquiring their cat, with BC owners generally knowing less about their cat (e.g., how it was born, and whether its parents had undergone appropriate pre-breeding testing). On a more positive note, over 70% of P and BC owners had seen both of their cat’s parents before buying it, compared to only 29% in the recent PAW Report [9].

## 5. Conclusions

Marked differences between cat type/breed were identified in this study in relation to acquisition approaches and procedures, including NP owners being younger than P and BC owners, as well as more NP owners being first-time owners compared to P and BC owners. Moreover, P and BC cats were more likely to conduct research prior to acquisition of their cat compared to NP owners, and owners of BC cats were more likely to score lower on ease of maintenance, and higher on cost compared to owners of NP and P cats.

Further research is required to determine how owner behaviour during cat acquisition may be improved in line with best practice, especially regarding irresponsible and potentially harmful acquisition methods. Furthermore, the findings of this study suggest that BC cat owners (and possibly other pedigree owners) are not fully aware of the potential health problems of their cats and/or underestimate their severity, jeopardising their cats’ health and welfare. This highlights the need for educating owners, breeders and veterinary professionals about responsible pet acquisition, conscientious breeding, BC-related disorders and cat welfare.

## Figures and Tables

**Table 1:** Respondent demographics.

Category	Sub-Category	Frequency	Percent	Category	Sub-Category	Frequency	Percent
Country	UK	589	43.1%		£150,000 or more	20	1.5%
	US	365	26.7%				
	Other	413	30.2%		Rather not answer/Unsure	267	19.5%
Age	18–24	221	16.2%	Marital Status	Single	340	24.9%
	25–34	393	28.7%		In a relationship	399	29.2%
	35–44	279	20.4%		Married	517	37.8%
	45–54	252	18.4%		Separated	20	1.5%
	55–64	148	10.8%		Divorced	71	5.2%
	65 or older	74	5.4%		Widowed	17	1.2%
Gender	Female	1195	87.4%		Other	3	0.2%
	Male	160	11.7%	Living Area	Urban	482	35.3%
	Other/Rather not answer	12	0.8%		Rural	323	23.6%
					Suburban	562	41.1%
Level of Education	Secondary education and below	537	39.3%	Residence	House/Semi-detached	981	71.8%
					Flat/Apartment	376	27.5%
	Higher Education	830	60.7%		Other	10	0.7%
Household Income	Less than £10,000	109	8.0%	Children (under 18)	No children	1058	77.4%
	£10,000–£24,999	255	18.7%		With children	309	22.6%
				Other pets other than cats	No	830	60.7%
	£25,000–£49,999	370	27%		Yes	537	39.3%
	£50,000–£74,999	193	14.1%		Dogs Other >1 type of pet	376 344 371	
	£75,000–£99,999	99	7.2%	First time cat owner	Yes	235	17.2%
	£100,000–£149,999	54	4.0%		No	1132	82.8%

**Table 2:** Feline demographics.

Category	Sub-Category		Frequency	Percent (of Total)
**Type/Breed**	Non-pedigree		882	64.5%
	Pedigree	Total	485	35.5%
		All BC breeds	169	12.4%
		Extreme BC breeds	80	5.9%
Age	0–3		533	39.1%
	3–6 years		352	25.82%
	6–9 years		204	14.96%
	9–12 years		124	9.09%
	12–15 years		76	5.57%
	15+ years		55	4.03%
	I do not know		19	1.39%
Sex	Female		679	49.7%
	Male		681	49.8%
	Other		7	0.5%
Neutered	Yes		1133	82.8%
	No		216	15.8%
	I do not know		18	1.3%
Outdoor access	No		483	35.41%
	Outdoor living		21	1.53%
	Unrestricted access to the outside at all times		215	15.76%
	Partial access to the outside		267	19.57%
	Access to a controlled outdoor area		264	19.35%
	Only on a harness		114	8.35%

All BC—all Brachycephalic breeds: British Shorthair, Persian, Burmese, Himalayan, Exotic Shorthair, Scottish Fold; Extreme BC—extreme Brachycephalic breeds: Persian and Exotic Shorthair.

**Table 3:** Characterisation of owners by cat type.

Variable	Sub-Category	% NP	% P	% BC	Variable	Sub-Category	% NP	% P	% BC
Country	UK	42.9	47.8	23.5	Children	No	75.2	82.3	77.6
	US	27.9	20.3	44.7		Yes	24.8	17.7	22.4
	Other	29.3	32.0	31.8	Other pets *	No	60.9	58.8	68.2
Age	18–24	21.5	5.5	10.6		Yes	39.1	41.3	31.8
	25–34	31.3	23.5	27.1		Dogs	27.8	28.3	21.2
	35–44	20.9	20.5	15.3					
	45–54	13.6	26.3	31.8	First time cat owner	No	78.5	91.5	87.1
	55–64	8.7	15.3	11.8		Yes	21.5	8.5	12.9
	65+	4.0	9.0	3.5	Cat’s age when acquired	0–1 years	75.5	86.5	64.7
Gender *	Male	11.1	14.5	4.7		1–3 years	11.3	4.5	12.9
	Female	87.8	85.3	94.1		3–6 years	6.9	4.3	12.5
Education	Secondary	40.4	37.3	37.6		6–9 years	1.8	2.3	5.9
	Higher	59.6	62.8	62.4		9+ years	2.8	1.5	8.2
Marital status *	Single	28.1	17.0	28.2		I don’t know	1.6	1.0	0
	In relation	32.1	25.3	17.6	Cat’s age currently *	0–3 years	33.3	52.5	34.1
	Married	33.3	47.3	40.0		3–6 years	27.4	22.3	24.7
	Separated	1.5	1.8	0		6–9 years	16.3	11.8	15.3
	Divorced	3.9	6.5	12.9		9–12 years	10.3	5.0	15.3
	Widowed	0.9	2.0	1.2		12–15 years	6.1	4.3	5.9
Living area	Urban	36.6	30.8	42.4		15+ years	4.4	3.0	4.7
	Rural	22.1	26.5	25.9	Cat’s gender *	Female	49.4	49.3	54.1
	Suburban	41.3	42.8	31.8		Male	50.0	50.5	44.7
Residence *	House	70.2	76.3	67.1	Neutered *	Yes	90.8	65.8	81.2
	Flat	29.0	23.3	31.8		No	7.5	33.8	17.6

* The categories ‘Other’/’Rather not answer’/’Unsure’ are not included in the table; NP—non-pedigree; P—pedigree; BC—brachycephalic.

**Table:** (a)

Feature	Median score* (Total—All Cats)	*χ*^2^ test (DF)/FET	*p*-adj.
Appearance	3	FET = 16.5	0.005
Behaviour	3	FET = 13.2	0.005
Companionship	3	FET = 22.8	0.005
Ease of maintenance	3	FET = 112.7	0.005
Health	3	FET = 17.8	0.005
Energy/exercise level	3	FET = 29.4	0.005
Costs	2	*χ*^2^(4) = 67.8	0.005

* Median score for a scale of 1–3 (1 is ‘poor’, 3 is ‘good’); FET—Fisher’s exact test.

**Table:** (b)

Feature	% NP			% P			% BC		
	1	2	3	1	2	3	1	2	3
Appearance	1.0	4.8	94.2	0.7	0.8	98.5	0	4.7	95.3
Behaviour	2.7	16.1	81.2	1.0	10.3	88.7	2.4	10.6	87.1
Companionship	2.5	11.1	86.4	1.5	3.8	94.8	1.2	7.1	91.8
Ease of maintenance	1.9	12.2	85.9	4.3	12.3	83.5	25.9	37.6	36.5
Health	2.7	10.3	87.0	4.3	5.3	90.5	3.6	17.9	78.6
Energy level	6.5	18.6	74.9	2.3	13.9	83.8	5.9	32.9	61.2
Costs	29.9	41.4	28.8	11.8	45.9	42.4	9.4	42.4	48.2

**Table:** (a)

Factor	Median Score * (Total—All cats)	*χ*^2^ test (DF)/FET	*p*-adj.
Appearance	2	*χ*^2^(4) = 270.0	0.005
Health	1	*χ*^2^(4) = 124.4	0.005
Companionship	2	*χ*^2^(4) = 312.3	0.005
Popularity	1	*χ*^2^(4) = 81.1	0.005
Celebrity ownership	1	Fisher’s exact test = 14.5	0.005
Costs	1	*χ*^2^(4) = 1.4	0.82
Ease of maintenance	1	*χ*^2^(4) = 109.9	0.005

* Median score is from a scale of 1–3, 1 is ‘little or no influence’, 3 is ‘strong influence’. FET—Fisher’s exact test.

**Table:** (b)

Factor	% NP			% P			% BC		
	1	2	3	1	2	3	1	2	3
Appearance	50.9	21.1	28.0	15.5	10.0	74.5	14.1	17.6	68.2
Breed is healthy	62.2	13.2	24.6	29.8	18.3	52	54.1	17.6	28.2
Companionship	59.0	15.1	26.0	15.5	8.3	76.3	20.0	14.1	65.9
Popularity	89.2	5.3	5.4	72.3	13.3	14.5	62.4	18.8	18.8
Celebrity ownership	97.5	1.4	1.1	93.8	2.0	4.3	94.1	2.4	3.5
Costs	72.7	15.1	12.2	75.0	14.3	10.8	75.3	11.8	12.9
Ease of maintenance	60.2	14.1	25.7	31.0	21.8	47.3	70.6	15.3	14.1

FET—Fisher’s exact test.

**Table 6:** Owners’ satisfaction with their cat by breed/type.

Satisfaction	% NP			% P			% BC		
	1	2	3	1	2	3	1	2	3
Owner satisfaction *	0.8	0.9	98.3	0.5	0.8	98.3	0	2.4	97.6
Would recommend breed/type **	Y	N	NS	Y	N	NS	Y	N	NS
	89.8	1.7	8.5	90.8	3.5	5.8	77.6	8.2	14.1

* Owner satisfaction: in general, how happy are you with your cat? 1—unhappy; 2—neither happy nor unhappy; 3—happy. ** Y—yes; N—no; NS—not sure

**Table 7:** Research prior to acquisition according to the cat typ.

Research		% NP	% P	% BC
Research Prior to acquisition	Research on breed/type	28.0	77.0	58.8
	No research	72.0	23.0	41.2
Source *	Online research/websites	67.3	77.0	83.3
	Talking to a breeder	9.1	70.5	53.3
	Talking to a vet professional	34.8	17.4	6.5

* Percentage shown for every group is for the choice—‘Used very much’.

**Table 8:** Where did you acquire your cat?

Source *	% NP	% P	% B
Charity Rescue/Shelter	37.5	6.5	16.5
Breeder	2.4	71.0	54.1
Found/Rescued by owner	19.4	4.5	3.5
Friend/Neighbour	27.7	5.3	11.8
Pet Shop	1.7	1.0	3.5
Website	5.0	2.0	4.7
Self-bred	1.7	8.0	2.4

*n* = 1367; * the category—‘Other’ is not included in the table.

**Table 9:** How did you find out about this breeder?

Source *	% P	% BC
Personal website	21.7	28.3
Gumtree/other websites	10.1	13.0
Social media	12.6	13.0
Breed club register	14.0	6.5
Recommendation from friends/family	9.8	10.9
Recommendation from another breeder/cat show	18.5	15.2

*n* = 332, * the category—‘Other’ is not included in the table.

**Table 10:** Owners’ behaviours when purchasing from breeders.

Survey Question	Answers	% P	% BC	Survey Question	Answers	% P	% BC
Where did you meet the breeder to see your cat?	At their home	77.3	78.3	Was there a waiting list for your cat?	Yes	31.5	26.1
	At your own home	2.4	0		No	53.1	60.9
	Other	20.3	21.7		I do not know	15.4	13.0
Did you see the mother and father of your cat?	Mother only	20.3	10.9	Did the breeder have a “lifetime returns policy” on their cats? * *χ*^2^ (4,*N* = 332) = 6.11, *p* = 0.047	Yes	50.3	37.0
	Father only	2.8	4.3		No	25.9	43.5
	Both	61.5	63		I do not know	23.8	19.6
	Neither	15.4	21.7	Did you ask to see health records of the mother and father of your cat?	Yes	41.3	32.6
Did you see the brothers and/or sisters of your cat (from the same litter)? * *χ*^2^ (2,*N* = 332) = 6.16, *p* = 0.046	Yes, all	62.6	43.5		No	58.7	67.4
	Yes, some	20.3	32.6	Were health records available for the mother and father of your cat? Fisher’s exact test = 10.6 *p*-value = 0.021	Only for the mother	1.0	2.2
	No	17.1	23.9		Only for the father	0	4.3
How was your cat born? Fisher’s exact test = 10.4 *p*-value = 0.01	Naturally	90.9	76.1		For both parents	65.7	52.2
	By elective caesarean	0.7	2.2		No	3.5	4.3
	By emergency caesarean	1.4	0		I do not know	29.7	37.0
	I do not know	7.0	21.7	Had the parents of your cat undergone any genetic testing before breeding?	Mother only	0	0
How long has the breeder been breeding? * Fisher’s exact test = 13.88 *p*-value = 0.006	Less than 5 years	10.8	8.7		Father only	0	0
	5–10 years	27.3	8.7		Both	53.8	43.5
	10–20 years	29.4	23.9		Neither	7.0	8.7
	Over 20 years	22.0	39.1		There are no genetic tests available for this breed	4.2	4.3
	I do not know	10.5	19.6		I do not know	35.0	43.5
Are the breeders of your cat involved in cat shows?	Yes	80.8	69.6	Had the parents of your cat undergone any infectious disease testing before breeding (e.g., FeLV/FIV)?	Mother only	0	0
	No	7.3	10.9		Father only	0.7	0
	I do not know	11.9	19.6		Both	66.8	52.2
How many litters do the breeders of your cat breed per year on average?	They do not breed their cat(s) every year	14.3	8.7		Neither	6.3	6.5
	At least one litter per year	58.4	54.3		I do not know	26.2	41.3
	I do not know	27.3	37.0	Had the parents of your cat undergone any tests (e.g., hip X-rays, heart scans) prior to breeding? Fisher’s exact test = 10.6 *p*-value = 0.011	Mother only	1.4	0
Did you visit any other breeders before the one you purchased your cat from?	Yes	26.6	30.4		Father only	0	0
	No	73.4	69.6		Both	33.9	13.0
Did you visit the breeder on more than one occasion prior to purchasing your cat?	Yes	47.2	39.1		Neither	18.9	17.4
	No	52.8	60.9		I do not know	45.8	69.6

* *p*-value was significant but subsequent post hoc tests did not show significant differences between the groups. FeLV—Feline Leukemia Virus; FIV—Feline Immunodeficiency Virus.

**Table 11:** Diagnosed health problems by cat type.

Health problem	% NP	% P	% BC
Heart and blood vessels	3.1	2.8	8.2
Glands	2.0	1.5	4.7
Digestive system	5.9	7.0	5.9
Skin and/or hair	6.6	5.0	10.6
Muscles and/or skeleton	3.5	3.3	5.9
Reproductive organs	0.8	0.5	1.2
The urinary system	10.7	5.0	15.3
Breathing	5.2	4.5	7.1
Eyes	6.8	7.0	20.0
Mouth and/or jaw	7.3	6.0	5.9

## Supplementary Materials

Supplementary File 1

## Appendix A

The survey was composed of seven sections and is sent as a separate document.
