# Supplementary material for: Motivation of Owners to Purchase Pedigree Cats, with Specific Focus on the Acquisition of Brachycephalic Cats
Source: Animals (Basel). 2019 Jun 27;9(7):394. doi: 10.3390/ani9070394 (PMC6680495; doi:10.3390/ani9070394)
Supplement: Supplementary File 1 [file animals-09-00394-s001.pdf]

What were your experiences when acquiring your cat?

About this survey

Privacy

Your privacy will be respected and all information you supply is confidential. By submitting this online questionnaire, you give permission for your data to be analysed and published (anonymously). Your personal information will be held and used in accordance with the Data Protection Act 1998 and will not be disclosed to any unauthorised person or body. Only the principal investigators in this study will have access to the data on the returned questionnaires. The data collected will be collated and stored at The University of Edinburgh (SRUC). However, if you change your mind part way through, you can leave the questionnaire at any point without submission of any data.

Only individuals aged over 18 years old are eligible to participate in this study. By proceeding, participants confirm that they are over 18 years old.

1. **I have read and understood the above explanation and am willing to allow my data to be used for this research project and any resulting publications. I hereby consent to participate in this study.** *Required*

- Yes

Thank you in advance for your participation.

About you

2. Country: What is your country of residence?

3. Age: What is yourage?

- 18-24
- 25-34
- 35-44
- 45-54
- 55-64
- 65 or older

4. Gender: What is yourgender?

- Male
- Female
- Other
- Rather not answer

5. Level of education: What is the highest of level education you have completed?

- Some high school
- High school graduate (or equivalent)
- Some college/university

- Undergraduate degree (or equivalent)
- Trade/technical/vocational training
- Some postgraduate work
- Postgraduate degree (Master’s)
- Ph.D. or medical degree
- Veterinary medicine degree

6. Household income: What was your total household income before taxes during the past 12 months?

- Less than £10,000
- £10,000-£14,999
- £15,000-£24,999
- £25,000-£34,999
- £35,000-£49,999
- £50,000-£74,999
- £75,000-£99,999
- £100,000-£149,999
- £150,000 or more
- Not sure
- Rather not answer

7. Marital status: What is your marital status?

- Single
- In a relationship
- Married
- Separated
- Divorced

- Widowed
- Other

Your home

8. **Living area: What type of area do you live in?**

- Urban
- Rural
- Suburban

9. **Residence: What kind of house do you live in?**

- Detached
- Semi-detached
- Terrace
- Flat / apartment
- Other

9.a. **If you selected Other, please specify:**

10. **How many rooms (in total) in your house?**

11. **How many people currently reside in your home?**

Men of 18 years, or more:	Please select

Women of 18 years, or more:	Please select
Children under 18:	Please select

12. **Do you have other pets in your household other than your cat/cats?** ***Tick all that apply***

- ☐ Dog(s)
- ☐ Rabbit(s)
- ☐ Hamster(s)/guinea pig(s)
- ☐ Bird(s)
- ☐ Reptile(s)
- ☐ No
- ☐ Other

12.a. **If you selected Other, please specify:**

About your cat(s)

In this section, some of the questions refer to just one cat. If you have multiple cats in your household, please answer these questions for your most recently acquired pedigree cat.
If you do not have any pedigree cats, please answer this question for your most recently acquired cat.

13. Are you a first-time cat owner?

- ◯ Yes
- ◯ No

14. How many cats do you currently own?

15. What type of cat(s) do you own? *Tick all that apply*.

- ☐ Non-pedigree cat
- ☐ Pure-breed
- ☐ Wild cat hybrid e.g. Bengal, Savanna, etc.
- ☐ I don't know / I'm not sure

16. If your most recently acquired cat is a pure-breed or wild cat hybrid, what is their breed?

- [ ] Abyssinian
- [ ] Asian
- [ ] American Bobtail
- [ ] American Curl
- [ ] American Shorthair
- [ ] American Wirehair
- [ ] Australian Mist
- [ ] Balinese
- [ ] Bengal
- [ ] Birman
- [ ] Bombay
- [ ] British Longhair
- [ ] British Shorthair
- [ ] Burmese
- [ ] Burmilla
- [ ] Chartreux
- [ ] Chausie
- [ ] Cornish Rex
- [ ] Cymric
- [ ] Devon Rex
- [ ] Donskoy
- [ ] Egyptian Mau
- [ ] Exotic Shorthair
- [ ] Havana Brown
- [ ] Himalayan
- [ ] Japanese Bobtail
- [ ] Khaomanee
- [ ] Korat
- [ ] Kurilian Bobtail
- [ ] LaPerm
- [ ] Maine Coon
- [ ] Manx
- [ ] Minuet
- [ ] Munchkin
- [ ] Nebelung
- [ ] Norwegian Forest
- [ ] Ocicat
- [ ] Oriental Shorthair
- [ ] Persian
- [ ] Peterbald
- [ ] Pixiebob
- [ ] Ragdoll
- [ ] Ragamuffin
- [ ] Russian Blue
- [ ] Savannah
- [ ] Scottish Fold
- [ ] Selkirk Rex
- [ ] Siamese
- [ ] Siberian
- [ ] Singapura
- [ ] Snowshoe
- [ ] Somali
- [ ] Sphynx
- [ ] Thai Lilac
- [ ] Tonkinese
- [ ] Toyger
- [ ] Turkish Angora
- [ ] Turkish Van
- [ ] Other

16.a. **If you selected Other, please specify:**

[ ]

Your previous experience with cat(s)

17. How many cats have you owned previously in your whole lifetime (Including those you own now)?

18. **If you have ever owned a different type of cat to those you currently own, what best describes their breed(s)?**

- [ ] Non-pedigree cat
- [ ] Pure-breed
- [ ] Wild cat hybrid e.g. Bengal, Savanna, etc.
- [ ] I have never owned any other type of cat

18.a. Please choose all previously owned pure-breeds and/or hybrids types of cat you have owned:

- [ ] Abyssinian
- [ ] Asian
- [ ] American Bobtail
- [ ] American Curl
- [ ] American Shorthair
- [ ] American Wirehair
- [ ] Australian Mist
- [ ] Balinese
- [ ] Bengal
- [ ] Birman
- [ ] Bombay
- [ ] British Longhair
- [ ] British Shorthair
- [ ] Burmese
- [ ] Burmilla
- [ ] Chartreux
- [ ] Chausie
- [ ] Cornish Rex
- [ ] Cymric
- [ ] Devon Rex
- [ ] Donskoy
- [ ] Egyptian Mau
- [ ] Exotic Shorthair
- [ ] Havana Brown
- [ ] Himalayan
- [ ] Japanese Bobtail
- [ ] Khaomanee
- [ ] Korat
- [ ] Kurilian Bobtail
- [ ] LaPerm
- [ ] Maine Coon
- [ ] Manx
- [ ] Minuet
- [ ] Munchkin
- [ ] Nebelung
- [ ] Norwegian Forest
- [ ] Ocicat
- [ ] Oriental Shorthair
- [ ] Persian

- [ ] Peterbald
- [ ] Pixiebob
- [ ] Ragdoll
- [ ] Ragamuffin
- [ ] Russian Blue
- [ ] Savannah
- [ ] Scottish Fold
- [ ] Selkirk Rex
- [ ] Siamese
- [ ] Siberian
- [ ] Singapura
- [ ] Snowshoe
- [ ] Somali
- [ ] Sphynx
- [ ] Thai Lilac
- [ ] Tonkinese
- [ ] Toyger
- [ ] Turkish Angora
- [ ] Turkish Van
- [ ] Other

18.a.i. **If you selected Other, please specify:**

19. If you have owned a different type of cat before, why did you acquire a cat of a different breed/type this time?

- ( ) This cat needed a new home
- ( ) Unhappy with the already owned breed
- ( ) Wanted another breed
- ( ) The same breed was not available
- ( ) Sudden availability of different breed
- ( ) I have never owned any other breed of cat
- ( ) Other

19.a. **If you selected Other, please specify:**

19.b. **Please explain why:**

About your cat

If you have multiple cats in your household, please answer ALL further questions for your most recently acquired pedigree cat.
If you do not have any pedigree cats, please answer this question for your most recently acquired cat.

20. **What is the name of your cat?** *Optional*

21. **How old was your cat when you acquired him/her (in years)?**

22. **How old is your cat now?**

23. **What is the sex of your cat?**

- ○ Female
- ○ Male
- ○ Other

24. **Has your cat been neutered?**

- ○ Yes
- ○ No
- ○ I don't know/not sure

25. **Do you intend to breed from your cat?**

- ○ Yes
- ○ No
- ○ Not sure yet/undecided

26. **Do you allow your cat outdoor access?**

- ○ Yes, my cat has free access to the outdoors
- ○ Yes, my cat has limited access to the outdoors (e.g. confined garden)
- ○ No

27. **Please rate each of these aspects of your cat, on a scale of 1-5 (*1 is very poor, 5 is very good*):**

	1. Very poor	2.	3.	4.	5. Very good
Appearance	☐	☐	☐	☐	☐
Behaviour	☐	☐	☐	☐	☐
Companionship	☐	☐	☐	☐	☐
Ease of maintenance	☐	☐	☐	☐	☐

Health	☐	☐	☐	☐	☐
Energy/Exercise levels	☐	☐	☐	☐	☐
Costs (1 is low costs, 5 is high costs)	☐	☐	☐	☐	☐

28. **In general, how happy are you with your cat?**

- ○ Very happy
- ○ Happy
- ○ Neither happy nor unhappy
- ○ Unhappy
- ○ Very unhappy

29. **Would you recommend this breed/type of cat to your friends and family?**

- ○ Yes
- ○ No
- ○ Not sure

29.a. **Please explain why:**

29.b. **Please explain why:**

29.c. **Please explain why:**

30. **Do you have any further comments with regards to your satisfaction/dissatisfaction with your cat's breed/type?**

Your research before getting your cat

31. **Did you carry out any research prior to acquiring your cat?**

- ○ Yes, on the breed/type of my cat
- ○ Yes, on cat ownership in general
- ○ Yes, on both breed/type and cat ownership
- ○ No

Your research before getting your cat

32. What sources did you use to research about your cat's breed/type and how much did you use these resources?

	Not at all	1. Used very little	2.	3.	4.	5. Used very much
Online research/websites	☐	☐	☐	☐	☐	☐
Talking to friends/family	☐	☐	☐	☐	☐	☐
Talking to a breeder	☐	☐	☐	☐	☐	☐
Talking to other cat owners	☐	☐	☐	☐	☐	☐
Talking to a vet/vet nurse/vet receptionist	☐	☐	☐	☐	☐	☐
Books	☐	☐	☐	☐	☐	☐
Cat magazines	☐	☐	☐	☐	☐	☐
Other	☐	☐	☐	☐	☐	☐

32.a. If you used *online sources/websites*, please specify:

32.b. If you used *other* resources, please specify:

33. **Prior to your research, how much did you feel you already knew about these topics in relation to your breed/type of cat?**

	1. I didn't know anything about this topic	2. I knew very little about this topic	3. I knew a moderate amount about this topic	4. I knew a sufficient amount about this topic	5. I knew a lot about this topic
Health	☐	☐	☐	☐	☐
Behaviour	☐	☐	☐	☐	☐
Lifespan	☐	☐	☐	☐	☐
Ease of maintenance	☐	☐	☐	☐	☐
Genetic problems	☐	☐	☐	☐	☐

34. **Following your research, how much do you feel you know about these topics in relation to your breed/type of cat?**

	1. I don't know anything about this topic	2. I know very little about this topic	3. I know a moderate amount about this topic	4. I know a sufficient amount about this topic	5. I know a lot about this topic
Health	☐	☐	☐	☐	☐
Behaviour	☐	☐	☐	☐	☐
Lifespan	☐	☐	☐	☐	☐
Ease of maintenance	☐	☐	☐	☐	☐
Genetic problems	☐	☐	☐	☐	☐

35. **Had you heard of any breed-related problems that affect the following parts of your cat's body before you acquired your cat? *Tick all that apply*.**

- ☐ Heart and blood vessels (e.g. heart disease)
- ☐ Glands e.g. the pituitary gland, thyroid glands (e.g. hyperthyroidism), pancreas (e.g. diabetes).
- ☐ Digestive system (including stomach, intestines, liver)
- ☐ Skin and/or hair
- ☐ Muscles and/or skeleton (e.g. joint problems, arthritis)
- ☐ Reproductive organs
- ☐ The urinary system (including the kidneys and bladder)
- ☐ Breathing (including nose, throat and lungs).
- ☐ Eyes (e.g. secretion, inflammation)
- ☐ Mouth and/or jaw
- ☐ I hadn't heard of any breed-related health problems
- ☐ I have a non-pedigree cat (not applicable)

Acquiring your cat

36. From where did you acquire your cat?

- ○ Breeder
- ○ Self bred
- ○ Friend/neighbour
- ○ Pet Shop
- ○ Charity Rescue Shelter (e.g. RSPCA, Blue Cross, etc.)
- ○ Website
- ○ Other

36.a. Please specify:

36.b. Please specify:

36.c. If you selected Other, please specify:

37. What was your motivation in acquiring this cat? *Tick all that apply*.

- ☐ Companionship
- ☐ As a companion for another animal
- ☐ Found/rescued
- ☐ Pre-cursor to children

- [ ] A family member/partner acquired it
- [ ] For the children
- [ ] For breeding
- [ ] Special needs (e.g. therapy animal)
- [ ] Space limitations but really wanted a pet
- [ ] Other

37.a. **If you selected Other, please specify:**

38. **If you paid to purchase your cat, how much did your cat cost (in £ GBP)?** *Optional*

39. **How much did the following factors influence your choice of cat?**

	1. Not an influence	2. Little influence	3. Moderate influence	4. Strong influence	5. Very strong influence
Appearance	☐	☐	☐	☐	☐
Childhood experiences (e.g. you grew up with this breed/type)	☐	☐	☐	☐	☐
Popularity of the breed/type	☐	☐	☐	☐	☐
Celebrity endorsement /ownership	☐	☐	☐	☐	☐

Good cat breed/type for children	☐	☐	☐	☐	☐
Good companion breed/type	☐	☐	☐	☐	☐
Cost (of the cat, plus potential vet bills, etc)	☐	☐	☐	☐	☐
This breed/type is generally healthy	☐	☐	☐	☐	☐
This breed/type is easy to take care of	☐	☐	☐	☐	☐
Recommended by a friend/family member	☐	☐	☐	☐	☐
Recommended by a veterinary professional (e.g. vet, vet nurse)	☐	☐	☐	☐	☐

40. **If you were to go back in time to before you acquired your cat, would you do anything differently?**

- ○ Yes
- ○ No

40.a. **What would you change?** ***Please tick all that apply*.**

- ☐ Levels of pre-purchase research
- ☐ Where I acquired my cat
- ☐ The breed/type I acquired
- ☐ I would not have acquired him/her

☐ Other

40.a.i. **If you selected Other, please specify:**

Acquiring your cat from a breeder

41. How did you find out about this breeder?

- ○ Personal website
- ○ Gumtree/otherwebsite
- ○ Social media
- ○ Newspaper advert
- ○ Breed club register
- ○ Recommendation from friends/family
- ○ Recommendation from another breeder
- ○ Other

41.a. If you selected O*ther website*, please specify:

41.b. If you selected Other, please specify:

42. Where did you meet the breeder to see your cat?

- ○ At their home
- ○ At your own home
- ○ Other

42.a. If you selected Other, please specify:

43. Did you see the mother & father of your cat?

- Mother only
- Father only
- Both
- Neither

44. Did you see the brothers and/or sisters of your cat (from the same litter)?

- Yes, all
- Yes, some
- No

45. How was your cat born?

- Naturally
- By elective (planned) caesarean section
- By emergency caesarean section
- I don't know

46. How long has the breeder been breeding?

- Less than 5 years

- 5-10 years
- 10-20 years
- Over 20 years
- I don’t know

47. Are the breeders of your cat involved in cat shows?

- Yes
- No
- I don’t know

47.a. If you selected Other, please specify:

48. How many litters do the breeders of your cat breed per year on average?

- They don’t breed their cat(s) every year
- At least one litter per year
- I don’t know

49. Did you visit any other breeders before the one you purchased your cat from?

- Yes
- No

50. Did you visit the breeder on more than one occasion prior to purchasing your cat?

- Yes
- No

51. Was there a waiting list for your cat?

- Yes
- No
- I don't know

52. Did the breeder have a “lifetime returns policy” on their cats?

- Yes
- No
- I don't know

53. Did you ASK to see health records of the mother and father of your cat?

- Yes
- No

54. Were health records AVAILABLE for the mother and father of your cat?

- Yes – only for the mother
- Yes – only for the father

- Yes – for both parents
- No
- I don’t know

55. Had the parents of your cat undergone any genetic testing before breeding?

- Mother only
- Father only
- Both
- Neither
- There are no genetic tests available for this breed
- I don't know

56. Had the parents of your cat undergone any infectious disease testing before breeding? e.g. FeLV / FIV?

- Mother only
- Father only
- Both
- Neither
- I don’t know

57. **Had the parents of your cat undergone any tests (e.g. hip X-rays, heart scans) prior to breeding? e.g. to check for hip dysplasia or heart disease.**

- Mother only
- Father only

- Both
- Neither
- I don’t know

Your cat's health and welfare

58. **In general, how healthy do you consider your cat to be over the course of his/her life, on a scale of 1-7 (*1 is very poor health, 7 is very healthy*)?**

	1. Very poor health	2.	3.	4.	5.	6.	7. Very healthy
Rank	☐	☐	☐	☐	☐	☐	☐

58.a. **If you rated the health of your cat as less than 7 (very healthy) please explain why you chose this:**

59. **How often has your cat visited a veterinary surgeon/received veterinary care per year, on average, since you acquired him/her?**

- Less than once a year
- Once or twice a year
- Three to five times a year
- More than five visits a year
- I have never taken my cat to the vet

60. **Why has your cat been taken to the vet? *Tick all that apply*.**

- ☐ Vaccinations
- ☐ Routine health checks
- ☐ For one short-term health problem

- ☐ For more than one short-term health problem
- ☐ For a long-term health problem
- ☐ For more than one long-term health problem
- ☐ For a physical injury
- ☐ For a behaviour problem

61. **Please estimate what you have paid in veterinary costs for your cat to date (in £ GBP). Please include costs covered by insurance:**

____

62. Do you have insurance for your cat?

- ○ No, I do not have insurance
- ○ Yes, but for accidents only
- ○ Yes, but I'm not sure of the details
- ○ Yes, but it is time-limited (e.g. for a year at a time, or up to a certain age)
- ○ Yes, to a maximum cost benefit per condition

62.a. In the amount of:

- ○ Up to £1000 per condition
- ○ Between £1000 and £4000 per condition
- ○ Between £4000 and £7000 per condition
- ○ For over £7000 per condition
- ○ Yes, but for a different amount than listed above

63. **Has your cat ever been diagnosed with any problems that affect any of the following parts of his/her body?** ***Tick all that apply*****.**

- ☐ Heart and blood vessels (e.g. heart disease or high blood pressure)
- ☐ Glands e.g. the pituitary gland, thyroid glands (e.g. hyperthyroidism), pancreas (e.g. diabetes).
- ☐ Digestive system (including stomach, intestines, liver)
- ☐ Skin and/or hair
- ☐ Muscles and/or skeleton (e.g. joint problems, arthritis)
- ☐ Reproductive organs
- ☐ The urinary system (including the kidneys and bladder)
- ☐ Breathing (including nose, throat and lungs).
- ☐ Eyes (e.g. secretion, inflammation)
- ☐ Mouth and/or jaw
- ☐ My cat has never been diagnosed with any of these problems

64. **To what extent do your cat's health problems negatively affect their quality of life?**

	My cat has no significant health problems with that part of the body	1. Have no effect on their quality of life	2.	3.	4.	5. Have a significant negative effect on their quality of life
Heart and blood vessels	☐	☐	☐	☐	☐	☐
Glands	☐	☐	☐	☐	☐	☐
Digestive system	☐	☐	☐	☐	☐	☐
Skin and/or hair	☐	☐	☐	☐	☐	☐

Muscles and/or skeleton	☐	☐	☐	☐	☐	☐
Reproductive organs	☐	☐	☐	☐	☐	☐
The urinary system	☐	☐	☐	☐	☐	☐
Breathing	☐	☐	☐	☐	☐	☐
Eyes	☐	☐	☐	☐	☐	☐
Mouth and/or jaw	☐	☐	☐	☐	☐	☐

65. Do you have any other comments on your cat's quality of life?

Thank you for completing and submitting this questionnaire.

Key for selection options

2 - Country: What is your country of residence?

- Afghanistan
- Albania
- Algeria
- American Samoa
- Andorra
- Angola
- Anguilla
- Antigua and Barbuda
- Argentina
- Armenia
- Aruba
- Australia
- Austria
- Azerbaijan
- The Bahamas
- Bahrain
- Bangladesh
- Barbados
- Belarus
- Belgium
- Belize
- Benin
- Bermuda
- Bhutan
- Bolivia
- Bosnia and Herzegovina
- Botswana
- Brazil
- Brunei
- Bulgaria
- Burkina Faso
- Burundi
- Cambodia

Cameroon
Canada
Cape Verde
Cayman Islands
Central African Republic
Chad
Chile
People's Republic of China
Republic of China
Christmas Island
Cocos (Keeling) Islands
Colombia
Comoros
Congo
Cook Islands
Costa Rica
Cote d'Ivoire
Croatia
Cuba
Cyprus
Czech Republic
Denmark
Djibouti
Dominica
Dominican Republic
Ecuador
Egypt
El Salvador
Equatorial Guinea
Eritrea
Estonia
Ethiopia
Falkland Islands
Faroe Islands
Fiji
Finland
France
French Polynesia
Gabon
The Gambia

Georgia
Germany
Ghana
Gibraltar
Greece
Greenland
Grenada
Guadeloupe
Guam
Guatemala
Guernsey
Guinea
Guinea - Bissau
Guyana
Haiti
Honduras
Hong Kong
Hungary
Iceland
India
Indonesia
Iran
Iraq
Ireland
Israel
Italy
Jamaica
Japan
Jersey
Jordan
Kazakhstan
Kenya
Kiribati
North Korea
South Korea
Kosovo
Kuwait
Kyrgyzstan
Laos
Latvia

Lebanon
Lesotho
Liberia
Libya
Liechtenstein
Lithuania
Luxembourg
Macau
Macedonia
Madagascar
Malawi
Malaysia
Maldives
Mali
Malta
Marshall Islands
Martinique
Mauritania
Mauritius
Mayotte
Mexico
Micronesia
Moldova
Monaco
Mongolia
Montenegro
Montserrat
Morocco
Mozambique
Myanmar
Nagorno-Karabakh
Namibia
Nauru
Nepal
Netherlands
Netherlands Antilles
New Caledonia
New Zealand
Nicaragua
Niger

Nigeria
Niue
Norfolk Island
Turkish Republic of Northern Cyprus
Northern Mariana
Norway
Oman
Pakistan
Palau
Palestinian territories, Occupied
Panama
Papua New Guinea
Paraguay
Peru
Philippines
Pitcairn Islands
Poland
Portugal
Puerto Rico
Qatar
Romania
Russia
Rwanda
Saint Barthelemy
Saint Helena
Saint Kitts and Nevis
Saint Lucia
Saint Martin
Saint Pierre and Miquelon
Saint Vincent and the Grenadines
Samoa
San Marino
Sao Tome and Principe
Saudi Arabia
Senegal
Serbia
Seychelles
Sierra Leone
Singapore
Slovakia

Slovenia
Solomon Islands
Somalia
Somaliland
South Africa
South Ossetia
Spain
Sri Lanka
Sudan
Suriname
Svalbard
Swaziland
Sweden
Switzerland
Syria
Taiwan
Tajikistan
Tanzania
Thailand
Timor - Leste
Togo
Tokelau
Tonga
Transnistria Pridnestrovie
Trinidad and Tobago
Tristan da Cunha
Tunisia
Turkey
Turkmenistan
Turks and Caicos Islands
Tuvalu
Uganda
Ukraine
United Arab Emirates
United Kingdom
United States
Uruguay
Uzbekistan
Vanuatu
Vatican City

Venezuela
Vietnam
British Virgin Islands
Isle of Man
US Virgin Islands
Wallis and Futuna
Western Sahara
Yemen
Zambia
Zimbabwe

10 - How many rooms (in total) in your house?
1
2
3
4
5
6
7
8
9
10+

11.1.a -
0
1
2
3
4
5
6
7
8
9
10+

11.2.a -
0
1

2
3
4
5
6
7
8
9
10+

11.3.a -

0
1
2
3
4
5
6
7
8
9
10+

14 - How many cats do you currently own?

1
2
3
4
5
6
7
8
9
10+

17 - How many cats have you owned previously in your whole lifetime (Including those you own now)?

1
2

3
4
5
6
7
8
9
10+

21 - How old was your cat when you acquired him/her (inyears)?
Between 0-1 year
1
2
3
4
5
6
7
8
9
10
11
12
13
14
15
16
17
18
19
20+
I don't know/not sure

22 - How old is your cat now?
Between 0-1 year
1
2
3
4

5
6
7
8
9
10
11
12
13
14
15
16
17
18
19
20+
I don't know/not sure
